# Progressive morphological changes and impaired retinal function associated with temporal regulation of gene expression after retinal ischemia/reperfusion injury in mice

**DOI:** 10.1186/1750-1326-8-21

**Published:** 2013-06-22

**Authors:** Byung-Jin Kim, Terry A Braun, Robert J Wordinger, Abbot F Clark

**Affiliations:** 1 The North Texas Eye Research Institute, University of North Texas Health Science Center, Fort Worth, TX 76107, USA; 2 Department of Pharmaceutical Sciences, College of Pharmacy, University of North Texas Health Science Center, Fort Worth, TX 76107, USA; 3 Departments of Biomedical Engineering and Ophthalmology & Visual Sciences, University of Iowa, Iowa City, IA 52242, USA; 4 Department of Cell Biology and Anatomy, Graduate School of Biomedical Science, University of North Texas Health Science Center, Fort Worth, TX 76107, USA

## Abstract

Retinal ischemia/reperfusion (I/R) injury is an important cause of visual impairment. However, questions remain on the overall I/R mechanisms responsible for progressive damage to the retina. In this study, we used a mouse model of I/R and characterized the pathogenesis by analyzing temporal changes of retinal morphology and function associated with changes in retinal gene expression. Transient ischemia was induced in one eye of C57BL/6 mice by raising intraocular pressure to 120 mmHg for 60 min followed by retinal reperfusion by restoring normal pressure. At various time points post I/R, retinal changes were monitored by histological assessment with H&E staining and by SD-OCT scanning. Retinal function was also measured by scotopic ERG. Temporal changes in retinal gene expression were analyzed using cDNA microarrays and real-time RT-PCR. In addition, retinal ganglion cells and gliosis were observed by immunohistochemistry. H&E staining and SD-OCT scanning showed an initial increase followed by a significant reduction of retinal thickness in I/R eyes accompanied with cell loss compared to contralateral control eyes. The greatest reduction in thickness was in the inner plexiform layer (IPL) and inner nuclear layer (INL). Retinal detachment was observed at days 3 and 7 post- I/R injury. Scotopic ERG a- and b-wave amplitudes and implicit times were significantly impaired in I/R eyes compared to contralateral control eyes. Microarray data showed temporal changes in gene expression involving various gene clusters such as molecular chaperones and inflammation. Furthermore, immunohistochemical staining confirmed Müller cell gliosis in the damaged retinas. The time-dependent changes in retinal morphology were significantly associated with functional impairment and altered retinal gene expression. We demonstrated that I/R-mediated morphological changes the retina closely associated with functional impairment as well as temporal changes in retinal gene expression. Our findings will provide further understanding of molecular pathogenesis associated with ischemic injury to the retina.

## Introduction

Retinal ischemia, often referred as “stroke of retina”, is an important cause of visual impairment in retinal vascular occlusion, diabetic retinopathy, glaucoma, and ocular trauma [1-5]. It is caused by a reduction of the retinal blood supply that decreases the delivery of oxygen and other nutrients to various retinal layers. Reperfusion of blood following ischemia is associated with oxidative stress and inflammatory responses [6]. In particular, resulting retinal ganglion cell (RGC) death is caused by a variety of cell death mechanisms including necrosis, apoptosis, necroptosis and autophagy after ischemia/reperfusion (I/R) injury [7-9]. To better understand the pathophysiological mechanisms associated with retinal I/R injury, several different experimental approaches have been designed in rodent models [8,10-12].

The “pressure-induced retinal I/R model” involves cannulation of the ocular anterior chamber followed by raising intraocular pressure above systolic blood pressure. After a specified period of time, the cannula is removed allowing restoration of retinal blood flow. This model has been used for the investigation of ischemia-derived ocular pathologies such as glaucoma and diabetic retinopathy [13,14]. This model currently is the most widely used method to study ocular diseases related to retinal ischemia.

Retinal damage due to I/R injury is associated with the loss of neurons, morphological degeneration of the retina, loss of retinal function, and ultimately vision loss [15-17]. Although degeneration times vary in different experimental conditions, I/R-induced injury and retinal degeneration is initially observed primarily in inner retinal layers [e.g. the inner plexiform layer (IPL) and inner nuclear layer (INL)] that are supplied by the central retinal artery, in contrast to the outer nuclear layer (ONL) that is generally less affected [18-21]. Differently from inner retinal layers, the choroid supplies blood and nutrients to the photoreceptors and ONL [22,23]. This structural difference may influence the initiation stage of I/R injury. Furthermore, morphological changes are often associated with functional impairment of the retina [24-26]. However, the correlation between morphological and functional changes with molecular mechanisms from different stages of pathogenesis associated with retinal I/R injury in retina is poorly characterized.

Several signaling pathways have been reported as key molecular events related to the degeneration or protection of RGCs and their axons in retinal I/R injury. Increased nuclear factor (NF)-κB p65 immunoreactivity was associated with retinal degeneration following retinal ischemia and reperfusion injury in mice [27], and inactivation of astroglial NF-κB promotes survival of retinal neurons following ischemic injury [28]. In addition to NF-κB, cyclooxygenease-2 appears to play a critical role in RGC death after transient ischemia [29]. Activation of Stat3 protects retinal ganglion cell layer neurons in response to transient retina ischemia in mice [30]. Several reports have indicated that inflammatory responses associated with the innate and adaptive immune system are major pathological processes in retinal I/R injury. These include toll-like receptor 4, complement component C3, tumor necrosis factor receptor, and surface molecule CD40 that are associated with functional impairment, retinal layer morphological changes, and/or loss of RGCs [20,31-33]. However, further studies are still required to define and better understand pathologic progression including temporal changes in retinal morphology, function, and molecular signaling.

In our study, we used a mouse model of pressure-induced retinal I/R injury and characterized morphological changes in retinal layers associated with retinal ERG functions. Using histological analysis and SD-OCT (spectral domain-optical coherence tomography) scanning, we showed progressive morphological changes of retinal layers at various times after I/R injury. In addition, we used scotopic electroretinography (ERG) to demonstrate functional deficits after I/R injury that corresponded to the morphological changes. We also profiled changes in retinal gene expression associated with I/R injury at various time points using cDNA microarray analysis to correlate the molecular mechanisms with morphological and functional changes.

## Material and methods

### Mice

Female C57BL6/J mice (8-10 weeks of age) were maintained in 12:12 light /dark cycle under optimal temperature and humidity controlled conditions. All studies were approved by University of North Texas Health Science Center’s Institutional Animal Care and Use Committee (IACUC) and complied with the ARVO Statement for the Use of Animals in Ophthalmic and Vision Research.

### Pressure induced retinal ischemia

Mice were anesthetized using a ketamine/xylazine cocktail (100/10 mg/kg). While anesthetized, mice were placed on a heating pad to prevent hypothermia. Mydfrin (Alcon, Inc. Fort Worth, TX) was topically administered to the test eye to dilate the iris. A 30-gauge needle connected to saline reservoir was inserted into the anterior chamber through the cornea of left eyes. Intraocular pressure was raised to 120 mmHg for 60 min. Retinal ischemia was confirmed by blanching of the retina using an ophthalmoscope. Contralateral right eyes served as controls. The needle was removed after 60 minutes to allow reperfusion of the retina. Tobrex (Alcon Inc. Fort Worth, TX) was topically administered to prevent ocular infection.

### Spectral domain-optical coherence tomography (SD-OCT)

Mouse retinas were scanned with an SD-OCT Ophthalmic Imaging System (Bioptigen Inc. Durham, NC). Briefly, mice were anesthetized using a ketamine/xylazine cocktail (100/10 mg/kg) and placed on the mouse holder to fix the mouse posture for scanning. The retina was scanned with rectangular scanning mode (1.2 mm diameter) consisting of 100 B scans/1000 A scans per B scan using InVivoVue software (Bioptigen Inc, Durham, NC). Superior, center and inferior images were imported and analyzed by ImageJ software (NIH) with four vertical calipers on each retinal layer (n = 4-5 mice per time point).

### Flash scotopic electroretinogram (ERG)

Mice were dark adapted for 16 hrs. After anesthesia with Ketamine/Xylazine (100/10 mg/kg), mice were placed in a Gantzfield light chamber on the LKC electroretinogram system (LKC Technologies Inc., Gaithersburg, MD) with temperature control (37°C). Amplitude and implicit times of ERG waveforms were measured at a series of flash intensities (-30, -20, -10, 0, 5, 10, 15 dB) (n = 9 mice per time point).

### Histology and cell counting in RGC layer

Eyes were harvested and fixed in neutral buffered 10% or 4% paraformaldehyde. After paraffin embedding, retinal cross sections were prepared (5 μm) followed by Hematoxylin-Eosin (H&E) staining for morphological observation of the retinal layers. Four retinal sections from ora serrata to ora serrata through the optic nerve head were chosen from each eye and the cells in the RGC layer were counted and averaged. Day 0 RGC counts from non-ischemic control eyes were set as 100%, and RGC cell counts in the rest of the eyes were compared to these controls.

### Analysis of retinal thickness

H&E stained whole retina or individual retinal layer thicknesses were measured using ImageJ software (NIH). Individual or whole (RGCL to ONL) layer thickness from 4 retinal cross-sections per eye were measured at quarterly points for each retinal cross-section and averaged. For SD-OCT images, layer thickness at 2 different distances from optic nerve head (~0.35 and 0.55 μm) was determined and averaged. Averaged retinal thickness was converted to the percentage of the thicknesses from day 0 non-ischemic contralateral eyes.

### RNA extraction and DNA microarray analysis

Total retinal RNA was extracted from control and I/R injured eyes. Retinas were collected and homogenized in Iso-RNA Lysis Reagent (5 PRIME Inc. Gaithersburg, MD). Total RNA was further extracted using an RNeasy Micro Kit (QIAGEN Science, Germantown, MD). RNA quality was controlled by determining RNA integrity number (RIN) using an Agilent 2100 Bioanalyzer (Agilent Technologies, Santa Clara, CA) with nano- or picochip systems. Total RNA with RINs >7 were selected and pooled from either control or experimental eyes at each time point for gene expression microarray analysis. Microarray hybridizations were performed at the University of Iowa DNA Core Facility. Briefly, 50 ng total RNA was converted to SPIA amplified cDNA using the WT-Ovation Pico RNA Amplification System, v2 (NuGEN Technologies, San Carlos, CA, Cat. #3302) according to the manufacturer’s recommended protocol. The amplified SPIA cDNA product was purified through a QIAGEN QIAquick PCR Purification column (QIAGEN Cat #28104) according to modifications from NuGEN. Five microgram samples were fragmented (average fragment size = 85 bases) and biotin labeled using the NuGEN FL-Ovation cDNA Biotin Module (NuGEN Technologies, Cat. #4200) per the manufacturer’s recommended protocol. The resulting biotin-labeled cDNA was mixed with Affymetrix eukaryotic hybridization buffer (Affymetrix, Inc., Santa Clara, CA), placed onto Affymetrix Mouse Gene 1.0 ST arrays (Part No. 901168) (Affymetrix Inc. Santa Clara, CA), and incubated at 45°C for 18 h with 60 rpm rotation in an Affymetrix Model 640 Genechip Hybridization Oven. Following hybridization, the arrays were washed, stained with streptavidin-phycoerythrin (Molecular Probes, Inc., Eugene, OR), and the signals were amplified with anti-streptavidin antibody (Vector Laboratories, Inc., Burlingame, CA) using the Affymetrix Model 450 Fluidics Station. Arrays were scanned with the Affymetrix Model 3000 scanner with the 7G upgrade and data were collected using the using the GeneChip operating software (GCOS) v1.4.

### Bioinformatic analysis

The 16 data CEL files for all 8 time points (0, 6 hr., 1, 3, 7, 14, 21 and 28 days) after I/R injury were imported into the Partek Genomics Suite 6.6 software (Partek Inc., Louis, MO) and normalized based on robust multi-array averaging (RMA). At each time point, the retinal I/R sample was compared to the contralateral control samples to calculate the microarray ratios and log_2_ fold change values. Using Excel, the selective filter of ≥1.5 fold change was utilized to identify up-regulated genes and ≤ -1.5 for the down-regulated genes per time point for the retina datasets. The genes were further analyzed using the publicly available bioinformatics software DAVID (Database for Annotation, Visualization and Integrated Discovery). Gene ontology (GO) based cluster analysis was performed to identify possible enrichment of genes (GO enrichment score calculated using a χ^2^ test) using filtered genes from each time point. The Fishers Exact p value is calculated by DAVID to identify GO enrichment based clusters, and p values <0.05 were considered to be significant in the enriched annotation category based on the Benjamini multiple test correction[34,35]. Clusters of genes were identified at specific time points and changes in gene expression were graphed temporally for each GO category.

### Real-time reverse transcription (RT)-polymerase chain reaction (PCR)

Complementary DNA (cDNA) was generated from the individual retinal RNA samples (n = 4-5) that were pooled for the microarray study. 2 μg of total RNA was used for reverse transcription (RT) at 25°C (10 min), 37°C (120 min) and 85°C (5 min) using a Multiscribe reverse transcriptase kit (Applied Biosystems, Life Technologies Corp., Grand Island, NY) and a PTC-100 thermal cycler (MJ Research Inc. Waltham, MA). Gene expression of *cryaa*, *cryba1*, *ccl12*, *c3* and *gapdh* was analyzed using Taqman gene expression assay primer sets (Applied Biosystems, Life Technologies Corp., Grand Island, NY) with Taqman Fast Advanced Master Mix (Applied Biosystems, Life Technologies Corp., Grand Island, NY) on a CFX96 real-time system (Bio-Rad, Life Science Research, Hercules, CA) with recommended thermal cycles by manufacturer (hold 50°C (2 min), hold 95°C (20 sec), 40 cycles of denaturation (3 sec) and annealing /extension (30 sec)). The expression of the housekeeping gene *gapdh* was used as an internal control to normalize target gene expression between samples. Differences in target gene expression were calculated using the following formula: ΔΔCT = ΔCT (target gene) – ΔCT (*gapdh*). The ΔΔCT value of cDNA amplification from the I/R eye was normalized to the control right eye. 0 hr was set as 1 and data from other time points were expressed as fold difference compared to 0 hr data.

### Immunohistochemistry

Sections of paraffin embedded retinas were subjected to antigen retrieval in citrate buffer (pH 6.0), and the slides were washed twice in PBS. Slides were blocked for 1 hr using SuperBlock Blocking Buffer (Thermo Scientific, Rockford, IL), and then incubated with mouse anti-Brn3a (1:50) (Cat # MAB 1585, EMD Millipore Corp. Billerica, MA), mouse anti-NeuN (1:1000) (Cat # MAB 377, EMD Millipore Corp. Billerica, MA), or rabbit anti-GFAP (1:200) (glial fibrillary acidic protein) (Abcam plc. cat.# ab7779, Cambridge, MA) for 16 hr. at 4°C, followed by washing and then incubation with goat anti-mouse antibody (1:500) or goat anti-rabbit antibody (1:500) conjugated with Alexaflour 488 (Cat.# A11001 or A11008, Invitrogen, Life Technologies, Grand Island, NY) for 1 hr. at room temperature. Cover slips were mounted using ProlongGold anti-fade reagent with DAPI (Molecular probes, Life Technologies, Grand Island, NY) for nuclear visualization. Images were acquired using a Nikon Eclipse Ti inverted microscope (Nikon Instruments Inc. Melville, NY) and a CRi Nuance FX multispectral imaging system (Caliper Life Sciences, Hopkinton, MA). Autofluorescence was subtracted using the Nuance 3.0 software.

## Results

### I/R induced retinal detachment after 3 and 7 days

**Figure 1:**
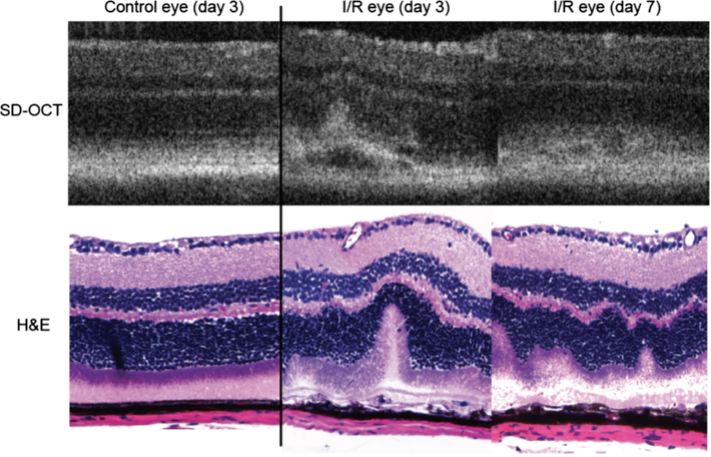
**I/R induced retinal detachment at day 3 and day 7 after injury.** SD-OCT scanning (upper panel) showed retinal detachment at the photoreceptor/RPE layer on days 3 and 7 after I/R injury. We confirmed I/R-induced retinal detachment using retinal H&E staining at the same points (lower panels). The contralateral eyes served as controls and had no detachment. Representative images of control or I/R retinas were selected from the same mouse used for SD-OCT scanning (n = 4-5) or H&E staining (n = 9-10).

During SD-OCT scanning, we first observed retinal detachment in all ischemic eyes 3 days after I/R injury. The detachment was also observed in I/R injured eyes at 7 days (Figure 1) and recovered by 14 days (data not shown). We morphologically confirmed similar retinal detachment 3 and 7 days after I/R injury by histological assessment of H&E stained retinas. In contrast, no morphological changes were observed in both SD-OCT and histological assessment from contralateral control eyes at day 3 from (Figure 1). The morphology of day 7-control eyes was not significantly different from day 3 eyes (data not shown).

### I/R injury induced progressive degeneration of inner retinal layers (IRLs) and decreased cell numbers in the retinal ganglion cell layer

**Figure 2:**
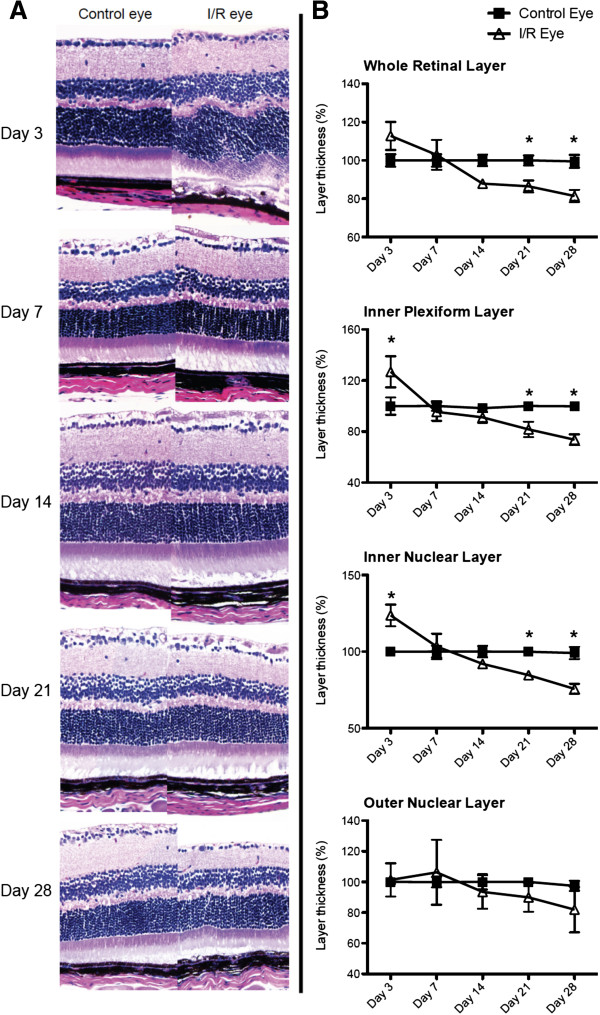
**I/R induced time-dependent degeneration of inner retinal layers*. ***Control and I/R mouse eyes were collected for histological assessment by H&E staining 3, 7, 14, 21 and 28 days after I/R injury with approximately 9-10 mice per time point. Whole retinal thickness (from NFL/RGCL to ONL) or sub-layer (IPL, INL and ONL) thickness at each time point was measured and statistically analyzed. Data represent mean ± standard error of percentages at each time point from both control and I/R eyes compared to the day 3-control eyes, which were set as 100%. For statistical analysis, two-way ANOVA (analysis of variance) was applied with the *Bonfferoni* post-hoc test. Asterisks indicate statistical differences (p < 0.05) between control and I/R eyes at each time point. (NFL/RGCL: nerve fiber layer/retinal ganglion cell layer, IPL: inner plexiform layer, INL: inner nuclear layer, ONL: outer nuclear layer).

**Figure 3:**
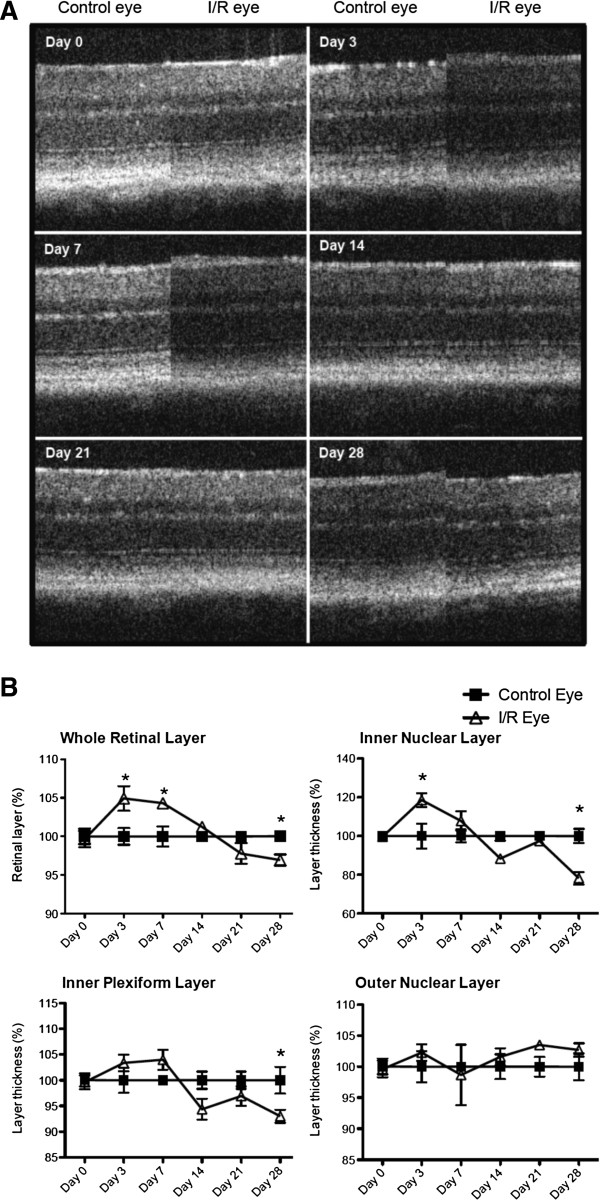
**Similar morphological changes were observed in I/R-injured retinal layers using non-invasive SD-OCT scanning.** Morphological changes in retinal layers were monitored using SD-OCT scanning. Time-dependent changes in retinal thickness were analyzed from whole retina (from RGCL to ONL) or retinal sub-layers (IPL, INL and ONL) at each time point. Data represent mean ± standard error of percentages at each time point from both control and I/R eyes compared to day 3-control eyes, set as a 100%. For statistical analysis, two-way ANOVA (analysis of variance) was applied followed by the *Bonfferoni* post-hoc test. Asterisks indicate statistical differences (p < 0.05) between control and I/R eyes at each time point. (RGCL: retinal ganglion cell layer, IPL: inner plexiform layer, INL: inner nuclear layer, ONL: outer nuclear layer).

Histological measurements of H&E stained retinas showed that I/R injury significantly (p < 0.05) increased retinal thickness of the inner retinal layers (IRLs), IPL and INL, at day 3 followed by progressive decreases in thickness after 14 days (Figure 2A and 2B). In addition, IRL thickness was significantly (p < 0.05) decreased at 21 and 28 days after injury compared to contralateral control eyes (Figure 2A and 2B). Layer thickness was decreased in only IRLs, IPL and INL, but not in the ONL (Figure 2B). We also observed similar trend changes using SD-OCT scanning. I/R injury significantly increased retinal thickness at early time points (3 and 7 days) followed by progressive decreases in retinal layer thickness over the remaining 28 days (Figure 3A and 3B). SD-OCT scanning also showed that the inner retinal layers (IPL and INL) were the sites of greatest damage induced by I/R injury (Figure 3A and 3B).

**Figure 4:**
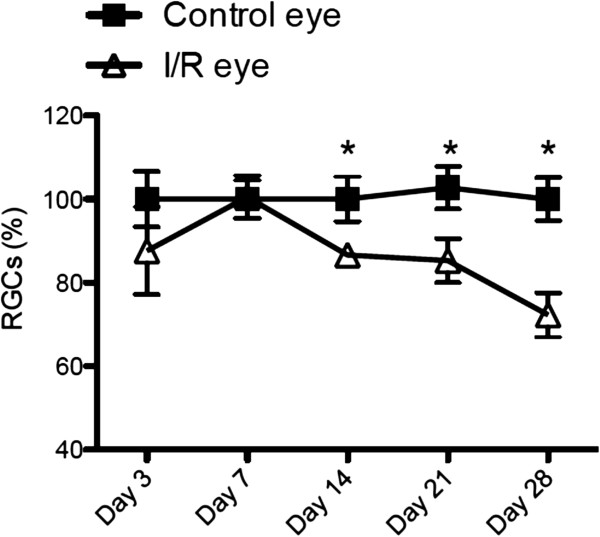
**Cell numbers in retinal ganglion cell layer (RGCL) progressively decreased after retinal I/R injury.** Total cell numbers in RGCL were counted from control or I/R-injured eyes (n = 9-10 per group) at each time point (3, 7, 14, 21 and 28 days) after I/R injury. Data represent mean ± standard error of cell number percentages at each time point from both control and I/R eyes compared to day 3-control eyes, set as 100%. Two-way ANOVA (analysis of variance) was applied with *Bonfferoni* post-hoc test for statistical analysis. Asterisks indicate statistical differences (p < 0.05) between control and I/R eyes at each time point.

**Figure 5:**
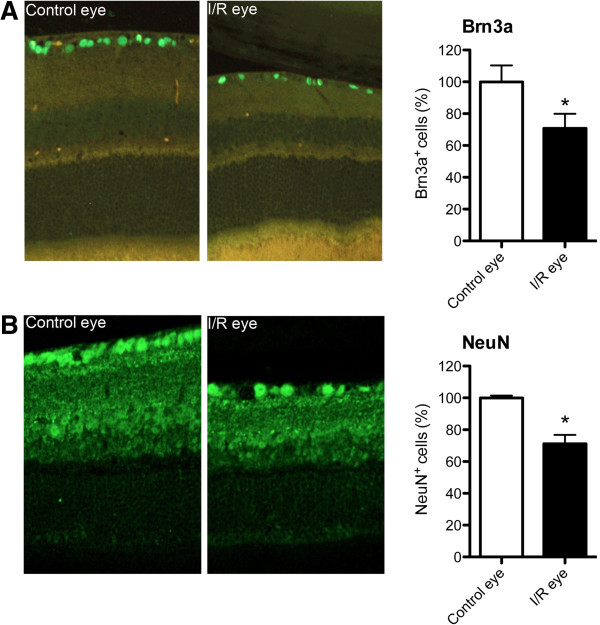
**Retinal I/R injury induced a significant decrease of Brn3a + or NeuN + positive RGCs 28 days post injury.** Retinas from 28 days post-I/R were stained for RGC markers Brn3a or NeuN. Total RGC numbers were counted from control or I/R-injured eyes (n = 4 per group). Data represent mean ± standard error of cell number percentages from both control and I/R eyes compared to non-injured control eyes, set as 100%. The student’s paired t-test was performed for statistical analysis. Asterisks indicate statistical differences (p < 0.05) between control and I/R eyes.

In addition, we counted cells in the RGC layer at 3, 7, 14, 21 and 28 days after I/R injury (Figure 4). Cell numbers in the RGC layer were significantly (p < 0.05) decreased compared to contralateral eyes beginning 14 days after I/R injury and further decreased over 28 days by approximately 30%, which correlates with morphological changes of I/R-injured retinal layers shown in Figures 2 and 3. We also confirmed our finding with RGC-specific Brn3a and NeuN immunohistochemical labeling. Our immunostaining data (Figure 5) also showed a significant 30% loss in RGCs 28 days post injury (p < 0.05). Since only approximately half of the cells in the RGCL are RGCs, these results suggest I/R damage to other RGCL cells (i.e. displaced amacrine cells).

### I/R induced retinal functional impairment beginning 3 days after injury

**Figure 6:**
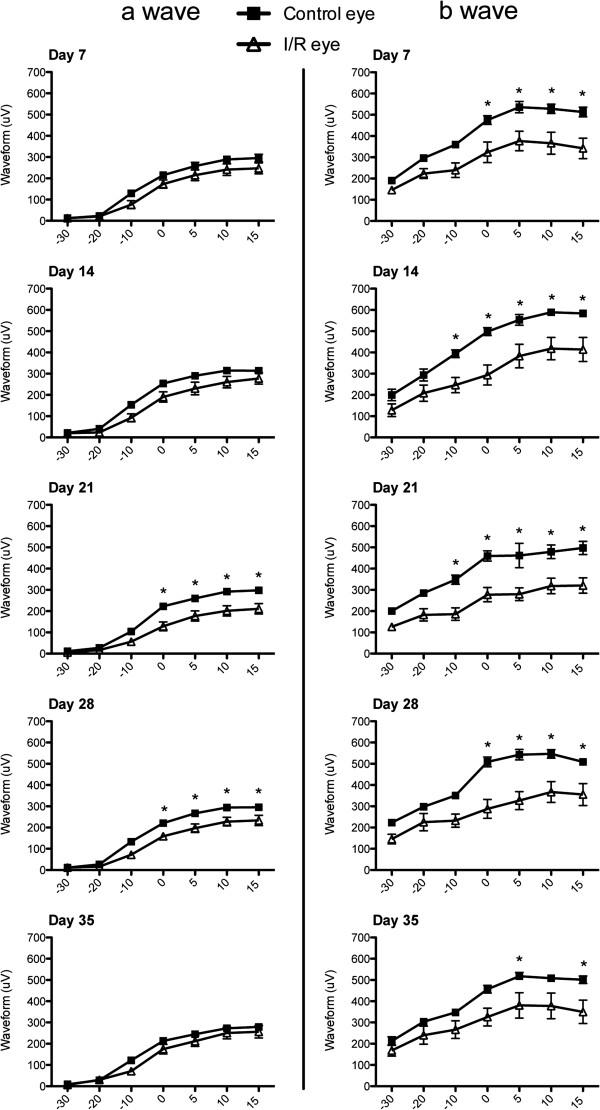
**I/R injury impaired retinal function.** Using electroretinography (ERG), the a- and b- wave amplitudes were measured from injured contralateral control eyes and I/R-injured eyes (n = 9 mice per group) under a series of light intensities (-30, -20, -10, 0, 5, 10, 15 dB) at 5 time points (7, 14, 21, 28 and 35 days) after I/R injury. Data represent mean ± standard error of a- or b-wave amplitudes (micro voltage) at each light intensity from both control and I/R eyes. Two-way ANOVA (analysis of variance) was applied with the *Bonfferoni* post-hoc test for statistical analysis. Asterisks indicate statistical differences (p < 0.05) between control and I/R eyes at each light intensity.

**Figure 7:**
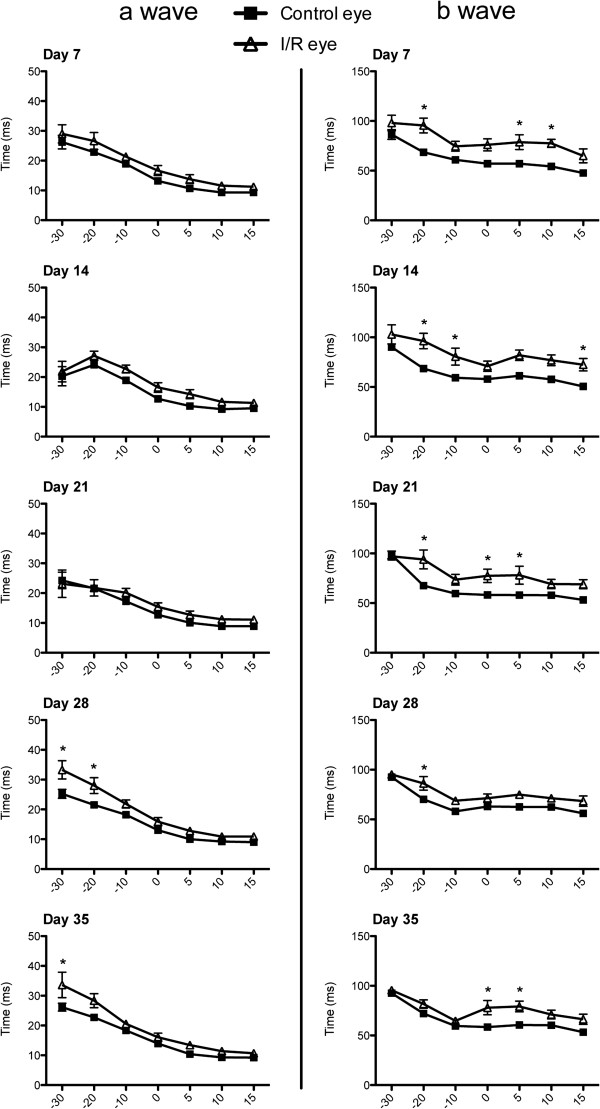
**I/R injury induced time latency in ERG waves.** The implicit times (i.e. latency times) of a- and b-waves were measured at varying light intensities during ERG recording (n = 9) at 7, 14, 21, 28 and 35 days after I/R injury. Data represent mean ± standard error of a- or b- wave implicit times at each light intensity from both control and I/R eyes. Two-way ANOVA (analysis of variance) was applied with the *Bonfferoni* post-hoc test for statistical analysis. Asterisks indicate statistical differences (p < 0.05) between control and I/R eyes at each light intensity.

In mouse eyes, 92 ~ 98% of the photoreceptors are rods, dominantly responsible for functional vision in this species [36,37]. Therefore, we used scotopic ERG to examine retinal function through amplitudes and implicit times of ERG a- and b-waves at 7, 14, 21, 28 and 35 days after I/R. No statistical changes were observed in a-wave amplitudes at 7 and 14 days after I/R injury, but amplitudes were significantly (p < 0.05) decreased at 21 and 28 days at higher light intensities (0, 5, 10 and 15 dB) in I/R injured eyes compared to contralateral control eyes. Interestingly, the a-wave amplitudes recovered at 35 day and did not show a statistical difference with the contralateral eyes. In contrast, b-wave amplitudes were significantly (p < 0.05) decreased by I/R injury at day 7 and continuously decreased (p < 0.05) over 35 days (Figure 6). We also measured changes in implicit times of a- and b-waves to monitor time latency. ERG a-wave implicit times did not significantly change at 7, 14 and 21 days after I/R injury but significantly increased at lower light intensities (-30 and -20 dB) by 28 and 35 days. In contrast, I/R injury significantly (p < 0.05) increased b-wave implicit times through all time points (7, 14, 21, 28 and 35 days) compared to contralateral eyes (Figure 7).

### I/R injury differentially altered expression of retinal genes at different time points

**Figure 8:**
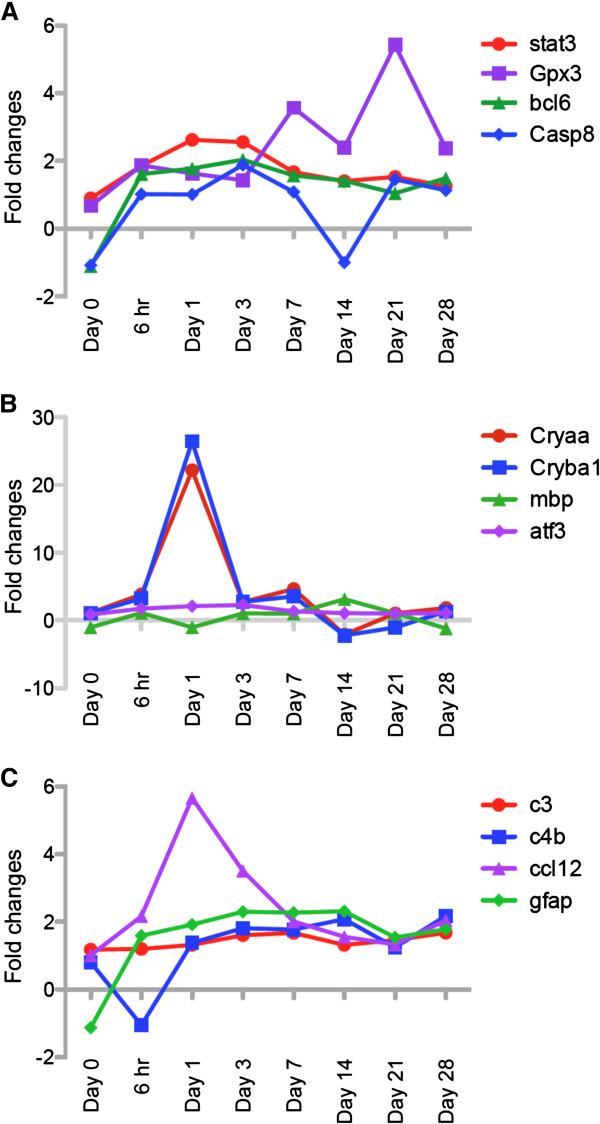
**I/R injury induced changes in retinal gene expression.** After normalizing to control eyes, changes of gene expression ratios in I/R-injured eyes were plotted based on log_2_ fold change values through 8 time points (0, 6 hr, 1, 3, 7, 14, 21 and 28 days) after I/R injury. Time dependent changes in gene expression for three signaling pathways are shown. Among various genes, changes of 12 genes were plotted based on their functional aspects such as signaling pathway related with (**A**) cell death/protection, (**B**) function as a molecular chaperone, and (**C**) inflammation.

We isolated total RNA from I/R-injured or contralateral retinas and analyzed gene expression profiles by microarray analysis at various time points (0, 6 hr., 1, 3, 7, 14, 21, 28 days) after I/R injury. Microarray data from I/R injured eyes were normalized based on the robust multi-array averaging (RMA) with contralateral control eyes using the Partek Genomics Suite 6.6 software. After selection of 1.5 or -1.5 fold (log_2_) up-or down-regulated genes, data were further analyzed using DAVID for gene clustering based on ontological identification using three categories: biological process (BP), molecular function (MF) and cellular function (CC). Up-regulated gene clusters are shown in Table 1. Gene clusters related with eye development were up-regulated at the earlier time points (6 hr., 1, 3, 7 days). This cluster included crystallin genes such as *cryaa* and *cryba1*. Up-regulation of these two genes was maximal (~20 fold log2 scale) at day 1 (Figure 8B), but expression was down-regulated at day 14 (Table 2). In contrast, gene expression of *mbp* (myelin basic protein) and *atf3* were consistently maintained through all time points. At all time points after I/R injury, gene clusters associated with inflammation such as inflammatory response, defense response or immune response were also up-regulated (Table 1). Complement genes *c3* and *c4b*, the chemokine gene *ccl12*, and *gfap* (glial fibrillary acidic protein) were identified in the gene clusters for inflammation (Figure 8C). Interestingly, *ccl12* expression was maximal at day 1, whereas the other genes (e.g. *gfap*) were gradually up-regulated and maintained throughout the time course. In addition, expression of apoptosis/cell death-related genes also increased, including *stat3*, *gpx3* (glutathione peroxidase 3), *bcl6*, and *casp8* (caspase 8) (Figure 8A).

**Table 1:** Gene ontology clusters in the mouse retina upregulated at various time points after I/R injury

**Time**		**Ontology**	**Enrichment score**	**P value (P < 0.05)**
0 day		No significant changes		
6 hr	BP	eye development	4.82	3.20E-04
		inflammatory response	4.49	2.20E-05
		apoptosis	1.76	8.40E-03
		blood Bessel morphogenesis	0.67	4.00E-02
	MF	structural constituent of eye lens	4.82	7.80E-09
		cytokine activity	3.15	3.80E-04
	CC	extracellular space	3.15	1.80E-04
1 day	BP	eye development	8.99	2.10E-09
		inflammatory response	3.51	2.20E-03
		cell death	1.37	1.10E-02
	MF	structural constituent of eye lens	8.99	1.80E-15
		transcriptional factor activity	1.52	1.80E-02
	CC	extracellular region part	2.71	1.30E-05
3 day	BP	inflammatory response	8.12	3.30E-08
		eye development	5.29	1.30E-04
		cell death	2.21	9.60E-04
	MF	structural molecule activity	5.29	3.30E-04
	CC	extracellular region	3.1	5.30E-04
7 day	BP	defense response	2.83	7.00E-09
		eye development	1.59	2.10E-02
	MF	peptide antigen binding	2.83	1.20E-02
	CC	extracellular region part	3.62	1.90E-04
		antigen processing and presentation	2.83	1.60E-03
14 day	BP	chemotaxis	3.2	3.90E-03
		cell-cell signaling	2.51	1.90E-03
		inflammatory response	1.96	6.50E-02
		cell death	1.73	3.40E-03
		tissue regeneration	1.51	5.60E-03
		myelination	1.51	4.90E-02
		neuron differentiation	1.35	3.80E-02
	MF	glutamate receptor activity	3.13	4.80E-04
	CC	extracellular region	6.58	6.60E-04
		neuron projection	4.31	3.30E-06
		synapse	3.13	1.20E-05
		postsynaptic membrane	3.13	2.80E-03
		gap junction	1.64	3.60E-02
21 day	BP	defense response	1.73	1.10E-02
		inflammatory response	1.73	3.00E-02
		hemopoiesis	1.3	4.00E-02
	MF	no significant changes		
	CC	extracellular region part	4.57	1.60E-07
		extracellular region	4.57	1.50E-06
		proteinaceous extracellular matrix	1.56	2.10E-04
28 day	BP	immune response	2.46	2.20E-04
		protein maturation	2.46	4.50E-02
		complement activation	2.46	7.10E-03
	MF	glycosaminoglycan binding	1.02	5.70E-02
	CC	extracellular region	3.48	3.90E-04
		proteinaceous extracellular matrix	1.36	3.70E-03

After normalization to control eyes, microarray data were filtered to select genes upregulated higher than 1.5 fold (log_2_). Data were further analyzed using DAVID (Database for Annotation, Visualization and Integrated Discovery) for gene clustering based on gene ontology to identify potential enrichment of genes. Gene clusters were identified into 3 categories, biological process (BP), molecular function (MF) and cellular component (CC) at 8 time points (0 hr, 6 hr, 1, 3, 7, 14, 21 and 28 days) after I/R injury.

**Table 2:** Gene ontology clusters downregulated in the mouse retina at various time points after I/R injury

**Time**		**Ontology**	**Enrichment score**	**P value (P < 0.05)**
0 day		No significant changes		
6hr	CC	extracellular region	1.64	3.10E-02
1 day		No significant changes		
3 day	BP	protein-DNA complex assembly	1.63	7.20E-03
	MF	DNA binding	1.63	2.10E-02
	CC	protein-DNA complex	1.63	8.30E-03
7 day		No significant changes		
14 day	BP	eye development	1.9	4.70E-02
	MF	structural constituent of eye lens	1.9	1.40E-03
21 day		No significant changes		
28 day	BP	G-protein coupled receptor protein signaling pathway	3.16	3.10E-05
	CC	integral to membrane	3.16	5.00E-02

Microarray data were filtered for selection of downregulated genes lower than -1.5 fold (log_2_) after normalizing with control eyes. Data were further analyzed using DAVID and clustered according to ontology with 3 categories (BP, MF and CC) at 8 time points (0 hr, 6 hr, 1, 3, 7, 14, 21 and 28 days) after I/R injury.

**Figure 9:**
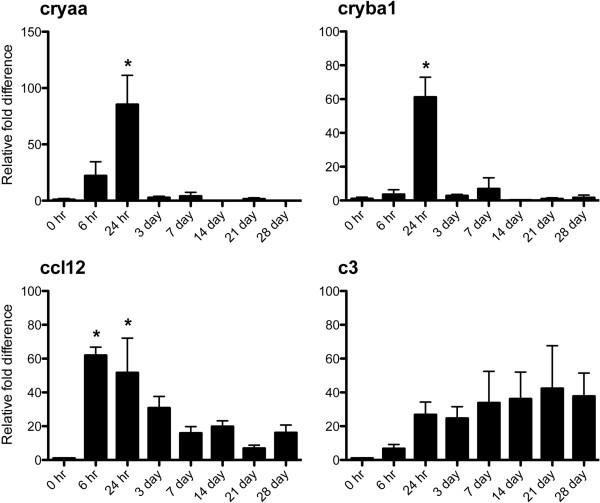
**Retinal mRNA expression of *cryaa*, *cryba1 *and *ccl12 *was significantly increased at 24 hr after I/R injury.** mRNA expression of *cryaa*, *cryba1*, *ccl12* and *c3* from I/R injured eyes was analyzed at 8 times points (0, 6hr, 1, 3, 7, 14, 21 and 28 days) by real time RT-PCR to confirm our microarray data. All data were normalized to *gapdh* expression followed by further normalizing to control eye data. Data represent mean ± standard error of relative fold increase of each gene compared to 0 hr expression set at 1. Asterisks indicate statistical differences (p < 0.05) between 0 hr and each time point.

Among various genes with altered expression, we selected *cryaa*, *cryba1*, *ccl12* and *c3* to validate our microarray data using real-time RT-PCT (Figure 9). In agreement with our microarray data, gene expression of *cryaa* and *cryba1* was significantly (p < 0.05) increased by ~84 and ~60 fold 24 hr after I/R injury. *ccl12* expression was also significantly (p < 0.05) increased ~50 fold at 6 and 24 hrs. after I/R injury. *c3* expression showed similar trend with microarray data for increased expression, but was not statistically different from controls due to variation of individual expression levels (Figure 9).

### I/R injury time-dependently increased retinal GFAP

**Figure 10:**
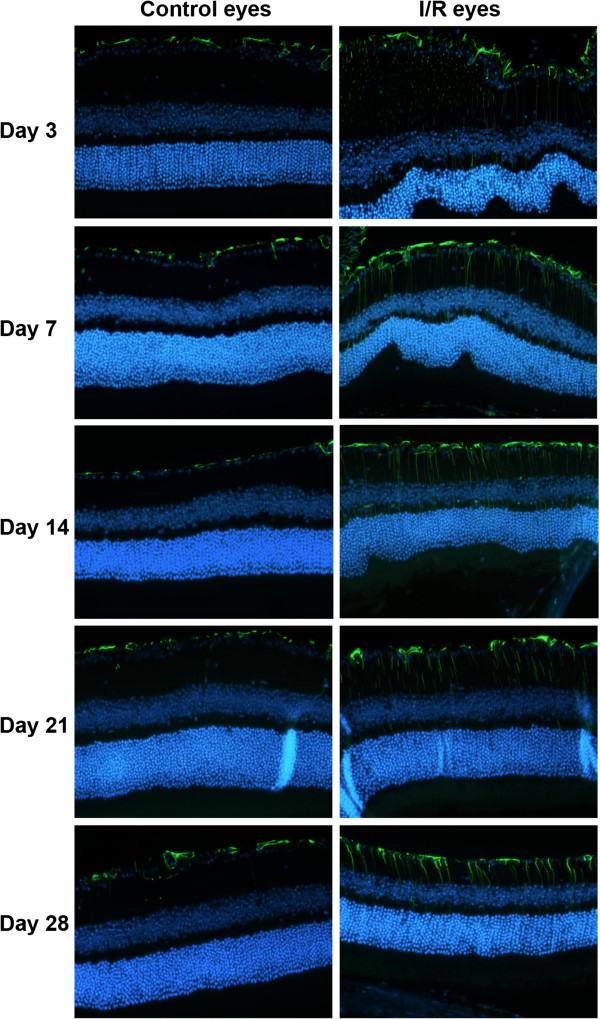
**I/R injury induced progressive gliosis in the retina.** Expression of retinal glial fibrillary acidic protein (GFAP) was monitored by immunohistochemistry at 5 time points (3, 7, 14, 21 and 28 days) after I/R injury. GFAP expression progressively increased through the retinal layers in I/R eyes compared to control eyes. Green fluorescence (Alexaflour 488) = GFAP. Nuclei were stained with DAPI (blue fluorescence).

In order to confirm increased retinal *gfap* mRNA expression, we evaluated GFAP immunostaining in the retinas of I/R injured and control eyes. Increased expression of GFAP is often used as a pathological gliosis marker associated with neuronal injury. In the retina, Müller cells and astrocytes are the major cell types that express GFAP, and its expression is increased in several ocular pathologies. In particular, gliosis in the Müller cells occurs through whole retinal layers under several inflammatory conditions [38-40]. As predicted by our gene array data, GFAP expression was up-regulated 3 days after I/R injury. GFAP expression was observed through the ONL at day 7, with maximal expression 14 days after I/R injury. After 21 days, the GFAP intensity decreased and was restricted to IRLs at 28 days after I/R injury (Figure 10). In contrast, there was no remarkable change in retinal GFAP expression of contralateral eyes at any time point (Figure 10). GFAP expression in control eyes was restricted to the RGCL, most likely due to astrocytes and/or Müller cell end feet. The increased GFAP staining in the I/R injured retinas shared morphological features of Müller cells.

## Discussion

Ischemic damage to different tissues such as brain and kidney share many similar pathologies [41,42]. In particular, retinal I/R results in neuronal degeneration associated with visual impairment and irreversible destruction of many layers of the structurally complex retina. Common morphological features in rodent models of retinal I/R include morphological degeneration of retinal layers, RGC death, and impairment of retinal function [18-21,24-26]. In order to discover overall pathological mechanisms, we observed pathological progression of I/R injury over 35 days, studying impairments in retinal morphology, function, and temporal changes in gene expression. There was a significant increase in thickness of inner retinal layers 3 days after I/R injury, most likely due to initial retinal edema. This histological finding was confirmed using non-invasive SD-OCT scanning. SD-OCT is now frequently used clinically and experimentally to detect morphological features of retina [43-47]. This technique allows live monitoring of the retina without mechanical invasion or damage. In contrast, traditional histological assessment is terminal and can introduce artifacts due to tissue processing. SD-OCT scanning allowed us to detect retinal detachment 3 days post injury in all mice, which was confirmed by histology (Figure 1). Both techniques confirmed time-dependent changes in retinal morphology after I/R injury.

Previous studies have suggested diverse molecular events promoting or attenuating I/R-induced retinal damage. A number of neuroprotective approaches have been tested in retinal I/R models. One major effort was to define protective mechanisms using pharmacological approaches. Ueda et al. reported that retinal neuronal injury occurred by both necrosis and apoptosis, which were inhibited by treatment with the cognition-enhancer, Nefiracetam [48]. Sun et al. also demonstrated that Cobalt protophorphyrin-induced Heme Oxygenase-1 attenuated I/R induced injury in retina [49]. The hypolopodemic drug Simvastatin also improved retinal ganglion cell survival in mouse model of retinal I/R injury [50]. More recently, Fujita et al. showed that pharmacological blocking of Angiotensin II type 1 receptor signaling produced neuroprotection via suppression of ROS production [51]. Genetic disruption of specific genes in mice has also been used to identify pathogenic and neuroprotective pathways. Aquaporin-4 null mice were protected from ischemia-induced retinal functional impairment and cell death [52]. Deletion of complement component C3 also induced retinal protection against I/R injury [32].

It is also important to examine molecular mechanisms associated with progression of retinal injury. cDNA microarray analysis is often used to provide extensive mRNA expression data [53-56]. Gene arrays provide profiles of functional gene clusters using a variety of bioinformatic approaches [34,35]. Youshimura et al. previously reported the temporal and spatial expression of immediate early genes in retinal neurons after retinal I/R injury [57]. Hollborn et al., also specifically identified inflammatory and immune-response-related genes activated in the early stage of experimental retinal detachment [58]. In addition, Kamphuis et al. evaluated changes in retinal gene expression following ischemic preconditioning [59]. In our study, we performed cDNA microarray analysis at 8 different time points after retinal I/R injury and verified their differential expression with real-time RT-PCR (Figures 8 and 9). Based on our gene clustering data, we observed temporal changes of several genes related with signaling pathways, structure/cellular stress and inflammation, based on their relation with ischemic diseases in the retina or other tissues. For example, Stat3 is protective in various ischemic diseases including retinal I/R injury [30]. Modulation of glutathione peroxidase (*gpx*) expression has been reported under ischemic environments in various tissues [60-63]. *bcl6* was originally known as a modulator of STAT-dependent interleukin-4 (IL-4) response in B cells [64]. *bcl6* is induced in circulated leukocytes after ischemic stroke, but its precise role in this condition is unknown [65]. Caspase8 is a major molecule in the apoptotic cascade involved in ischemia-induced cell death [66,67]. Although crystallins were originally known as structural proteins in lens [68-70] crystallins also are molecular chaperones structurally similar to small heat shock protein (hsp) with the ability to prevent protein aggregation [70-73]. Altered expression of the crystallins has been observed in various ocular diseases such as diabetic retinopathy, uveitis and glaucoma [74-78]. However, no clear changes in crystallin expression has been shown previously in retinal I/R injury.

One of the most interesting changes was the up-regulation of various inflammatory genes including *c3*, *c4b*, *ccl12*, and *gfap* (Figures 8, 9, 10). In particular, several researchers discovered that complement components play important roles in eye development and ocular pathology such as glaucoma [79]. In addition, genetic disruption of *c3* protected mice against retinal I/R injury [32]. Both *c3* and *c4b* genes encode C3 and C4b proteins, essential for the classical complement cascade [80,81]. Expression of these proteins is temporally regulated and may play differential roles at different times during I/R injury. Retinal GFAP expression is mainly observed in Müller cells during retinal injury [39,82,83]. Like other glial cells in neuronal system, Müller cells play a pivotal role to maintain retinal neuron homeostasis, such as scavenging neurotransmitter/waste products, supplying energy for retinal neurons, and other protective and maintenance roles for neurons [84,85]. Under pathological conditions, Müller cells are activated, undergoing functional and morphological changes associated with gliosis [39,82]. Hirrlinger et al. demonstrated that transient retinal ischemia in mice induced Müller cell gliosis accompanied by altered protein expression and changes in membrane properties [86]. Our data provide further support for Müller cell-dependent retinal gliosis, involving changes in gene and protein expression as well as Müller cell morphological changes that correlate with progression of I/R injury.

As previously mentioned, retinal thickness, especially in the inner retina, significantly increased within 3 days of retinal I/R injury. It is likely that this increased retinal thickness was due to retinal edema. Others have shown that the increased retinal thickness in retinal ischemia is due to retinal edema, which may be mediated by ET-1 in endothelial cells [12]. Retinal edema plays a major role in the pathogenesis of other types of retinal injury, including retinal vessel occlusion and diabetic retinopathy. In addition, progressive retinal degeneration following edema in our model was also strongly correlated with significant cell loss in the RGCL, including RGC and displaced amacrine cells. Immunohistological assessment using RGC specific antibodies to Brn3a and NeuN showed RGC loss induced by I/R injury. Interestingly, a similar cell loss ratio from histology data (~30%) was also shown in our immunohistochemistry data (~30%) at 28 days after I/R injury. These data suggest that I/R injury caused cell loss of both RGC and displaced amacrine cells in the RGCL. In support, Kim et al., previously showed that retinal I/R injury induced apoptotic cell death to both RGCs and displaced amacrine cells [87].

One of our novel observations is I/R-induced retinal detachment (Figure 1). Retinal detachment is a major cause of vision loss in various ocular pathologies, including age-related macular degeneration (AMD) [88-90]. We first observed the retinal detachment in all ischemic eyes by SD-OCT scanning at days 3 and 7 after I/R injury, but this detachment disappeared at 14 days. We took advantage of SD-OCT scanning to monitor the real-time morphological status of the retina without sacrificing mice. Interestingly, Zeng et al. developed a novel mouse model of retinal detachment using a similar cannulation method [91]. In contrast to our data, retinal detachment completely recovered within 24 hrs in their model. In support, Uckermann et al. also suggested that transient retinal ischemia in rabbits can cause exudative detachment of the retina through days 3 and 8, which was accompanied by changes in Müller cell K^+^ conductance [92]. They suggested fluid-mediated retinal detachment as a novel ischemia-mediated damage to the ONL. Our findings also support retinal detachment as another potential pathologic mechanism for temporal retinal dysfunction and degeneration. Our ERG a-wave data, which is associated with outer retinal photoreceptor function, also support this finding (Figure 6). Retinal detachment was associated with ERG a-wave amplitude deficits from days 21-28 after I/R injury. Interestingly, a-wave amplitudes recovered at 35 days, correlating with recovery of retinal detachment. These results suggest that early retinal detachment causes delayed outer retina ERG deficits that are reversible after retinal reattachment. Therefore, retinal detachment in the mouse model of retinal I/R may contribute to overall ischemia-mediated retinal damage.

Interestingly, all of our data showed strong temporal correlations. We detected thickening of the whole retina, especially the inner retina, 3-7 days after I/R injury. Fourteen days post I/R injury, four significant changes simultaneously occurred including: (1) decreased inner retinal layer thickness, (2) significant loss of cells in RGC layer, (3) significant changes in gene expression profiles, and (4) increased GFAP immunostaining (gliosis) throughout entire retina. Functional impairment (i.e. decreased ERG responses) began 7 days after I/R injury, suggesting decreased retinal function was due to early retinal edema and/or damaged retinal cells prior to morphological degeneration. Our findings are the first to demonstrate temporal morphological changes accompanied with functional and molecular changes associated with progression of retinal I/R injury.

In conclusion, transient I/R induced morphological changes mainly in the inner retina that were strongly associated with functional impairment as well as temporal changes in retinal gene expression. Our data also indicated that retinal detachment was induced by retinal ischemia in the early stages of injury. Our characterization of temporal retinal changes produced by retinal ischemia will lead to a better understanding of molecular pathogenesis associated with this injury as well as suggest novel therapeutic approaches to mitigate this retinal damage. Future studies will identify cellular and molecular mechanisms associated with I/R damage to the optic nerve and visual axis in the brain. This will lead to the discovery of new neuroprotective strategies and agents for the treatment of the retina, optic nerve, and visual axis in the brain associated with retinal I/R injury.

## Competing interests

The authors declare that they have no competing interests.

## Authors’ contributions

BK performed all experiments except microarray procedure and analyzed all data. BK also drafted manuscript including text and figures. TB participated in the microarray studies. RW provided guidance in the design and coordination of the study. AC designed and coordinated the study and finalized manuscript. All authors read and approved the final manuscript.
